# Supplementary material for: Individuals with ventromedial frontal damage display unstable but transitive preferences during decision making
Source: Nat Commun. 2022 Aug 13;13:4758. doi: 10.1038/s41467-022-32511-w (PMC9376076; doi:10.1038/s41467-022-32511-w)
Supplement: Supplementary file 1 — Supplementary Information [file 41467_2022_32511_MOESM1_ESM.pdf]

Supplementary Information for

Individuals with ventromedial frontal damage display unstable but transitive preferences during decision making

Authors: Linda Q. Yu, Jason Dana and Joseph W. Kable
Corresponding author:
Linda Q. Yu
linda_yu@brown.edu

This PDF file includes:

Supplementary methods
Supplementary discussion
Fig. S1
Supplementary references

Supplementary methods

Stimulus norming. The artwork stimuli were paintings that were rated highly by participants in (1). The set B stimuli consisted of 5 paintings by Monet, which were all within the top 20 most highly rated paintings by those subjects. We selected Monet as he was the artist that occurred most frequently in the top 20 rated paintings of (1). The 5 selected paintings were roughly similarly preferred (i.e., chosen with close to the same frequency in pair-wise choices across the whole sample) in a sample of 107 participants recruited from Amazon Mechanical Turk. Set A consisted of paintings of the similar style/era (Impressionist, Romantic periods) in the top 40 ranked paintings of the (1) stimuli set.

The chocolate bars were from five brands (Lindt, Godiva, Ghirardelli, Dove, and Cadbury). We selected five brands that were roughly similarly preferred across the population. These brands were being sold for similar prices, were rated similarly on a seven-point scale by a sample of 103 participants from Amazon Mechanical Turk (mean rating = 5.76), and were selected at roughly similar frequencies in pair-wise choices across another sample of 101 Mechanical Turk participants. Milk chocolate bars from each of the 5 brands were in set B, while dark chocolate and dark chocolate almond bars from each brand were in set A. The stimuli consisted of publicly available pictures of the front side of the chocolate bar packaging.

We used sixteen gambles of equal expected value ($8.80). The stimuli consisted of a pie chart showing the probability of winning, with text on top indicating both the cash amount to be won and the probability of winning. The five set B gambles were the “Cash II” set in (2), which used contemporary monetary equivalents of the (3) five gamble set. The probabilities were 28%, 32%, 36%, 40%, and 44%. Set A consisted of 11 other gambles with the same expected value (probabilities of 8%, 17%, 25%, 33%, 42%, 50%, 58%, 67%, 75%, 83%, 92%).

Sensitivity of probabilistic tests. We performed several simulations to determine the sensitivity of tests of the mixture model, i.e., the rate at which this test would declare different forms of random or heuristic-based choice to be intransitive. First, following (2), we randomly picked a choice probability for every pair from a uniform distribution (from 0 to 100%). As previously shown in that paper, only about 5% of the choice datasets simulated in this manner satisfy the triangle inequalities. That is, only 5% of the possible set of choice proportions for 10 pairs/5 stimuli satisfy the mixture model.

Second, we simulated an intransitive chooser who has an entirely consistent preference within each pair (i.e., choosing A 100% of time when it is paired with B) that is unconstrained by any higher order transitive structure (i.e., the preference in each pair is independent from that of all other pairs). This type of intransitive chooser only satisfies the triangle inequalities about 12% of the time for choice proportions for 10 pairs/5 stimuli.

Third, we simulated an intransitive chooser using the lexicographic semiorder heuristic (3). This heuristic is easiest to demonstrate with the gambles stimulus set. Following (3), we defined our lexicographic semiorder rule as follows: if two gambles are adjacent (i.e., next to each other in the set in terms of probabilities/payouts), always choose the gamble with the higher payout (amount); for all other (non-adjacent) gamble pairs, always select the gamble with the higher probability. Such a chooser would never satisfy the triangle inequalities in our dataset. Together, the first three sets of simulations show that our tests of mixture model are very sensitive to different forms of intransitive choice.

Supplementary Discussion

Additional effects on DDM parameters. As described in the main text, we performed a mixed ANOVA to test the effects of category (artworks, brands, gambles) and group on the DDM parameters. For the variability term, we found main effects of group ($F(2, 37) = 6.25$, $p = 0.005$) and category ($F(2,4) = 12.85$, $p < 0.001$), but no category x group interaction ($F(4,74) = 1.09$, $p = 0.37$). The main effect of group, as already explored in the main text, was driven by a higher variability term in the VMF group. The main effect of category was driven by a higher variability term in the artworks category (mean = 0.11) compared to both the brands (mean = 0.09) [$t(39) = 3.54$, $p = 0.001$] and gambles categories (mean = 0.08) [$t(39) = 3.96$, $p = 0.0003$)]. This result suggests that preferences in the artworks category were noisier across all participants.

For non-decision time, we found a significant main effect of category ($F(2,74) = 11.22$, $p < 0.0001$), but no significant effect of group, nor a significant category x group interaction ($F(4,74) = 0.938$, $p < 0.45$). The main effect of category was driven by a longer non-decision time in the gambles category (mean = 0.60) compared to both the artworks category (mean = 0.35, $t(39) = -3.59$, $p < 0.0001$) and the brands category (mean = 0.36, $t(39) = 3.49$, $p = 0.001$). This result may be due to the fact that the gambles stimuli required more reading than the other two categories.

There were no significant effects of category, nor significant category x group interactions, for any other parameters in the DDM.

Possible correlates of choice cycles. We found that three individuals with VMF damage exhibited a significantly greater number of choice cycles as individuals, compared to healthy controls. However, we did not find any particular differences between these three individuals, who showed the largest effects, and others in the VMF group. The overlap of the lesions of these three individuals is depicted in **Figure S1**. Within the VMF group, the total number of choice

cycles (i.e., across all three categories) was not significantly correlated with overall lesion size (in cc's; rho = -0.13, p = 0.67), or with lesion volume within a vmPFC mask defined based on value effects in fMRI studies (4) (rho = -0.06, p = 0.83).

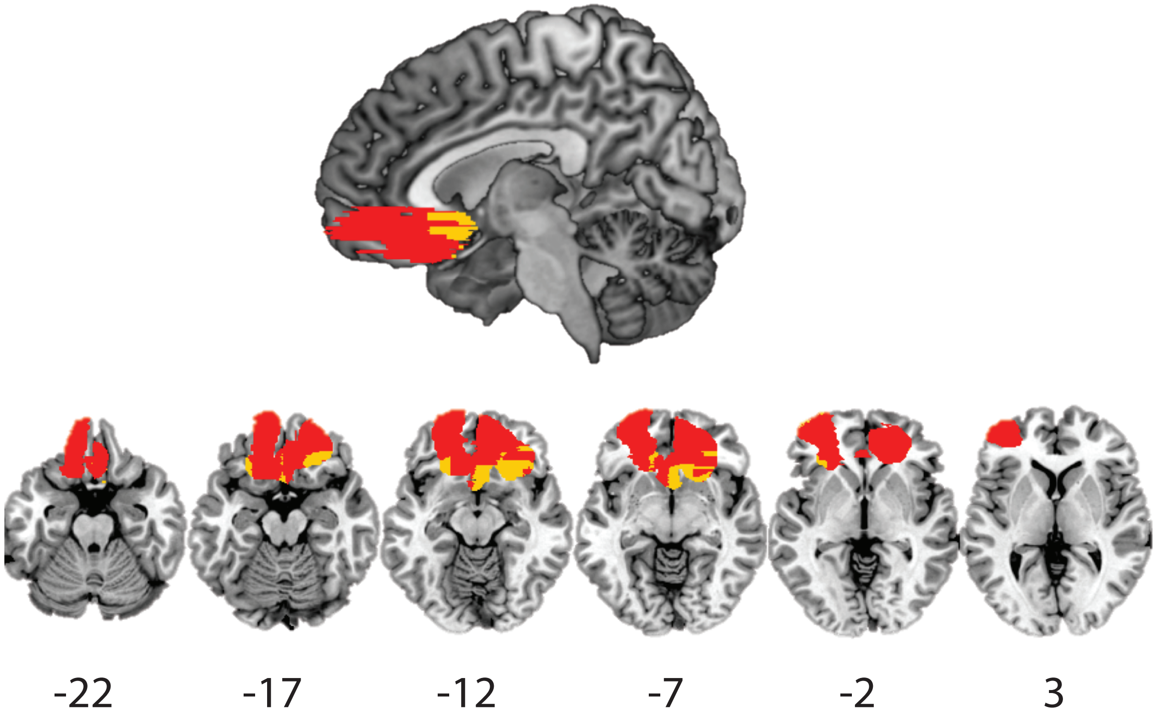

Figure S1: Lesion tracings of the three individuals with VMF damage who had significantly more cyclical choices compared to healthy control subjects at the individual level, as determined by case-control t-tests. Red denotes areas where at least one of these subjects had damage; yellow denotes the areas where at least one of these subjects had damage *outside* of all other subjects. There was very little overlap in lesions across the three subjects (only maximally two out of three and only in a small number of voxels). Numbers below axial slices indicate the MNI z-coordinates.

Supplementary references

1. A. R. Vaidya, L. K. Fellows, Testing necessary regional frontal contributions to value assessment and fixation-based updating. *Nature Communications* **6**, 10120 (2015).
2. M. Regenwetter, J. Dana, C. P. Davis-Stober, Transitivity of preferences. *Psychological review* **118**, 42-56 (2011).
3. A. Tversky, Intransitivity of preferences. *Psychological review* (1969).
4. O. Bartra, J. T. McGuire, J. W. Kable, The valuation system: a coordinate-based meta-analysis of BOLD fMRI experiments examining neural correlates of subjective value. *Neuroimage* **76**, 412-427 (2013).
