# Supplementary material for: Individuals with ventromedial frontal damage display unstable but transitive preferences during decision making
Source: Nat Commun. 2022 Aug 13;13:4758. doi: 10.1038/s41467-022-32511-w (PMC9376076; doi:10.1038/s41467-022-32511-w)
Supplement: Supplementary file 3 — Reporting Summary [file 41467_2022_32511_MOESM3_ESM.pdf]

nature research

Corresponding author(s): Linda Q. Yu

Last updated by author(s): 07/14/2022

Reporting Summary

Nature Research wishes to improve the reproducibility of the work that we publish. This form provides structure for consistency and transparency in reporting. For further information on Nature Research policies, see our Editorial Policies and the Editorial Policy Checklist.

Statistics

For all statistical analyses, confirm that the following items are present in the figure legend, table legend, main text, or Methods section.

n/a	Confirmed	
☐	☒	The exact sample size (n) for each experimental group/condition, given as a discrete number and unit of measurement
☐	☒	A statement on whether measurements were taken from distinct samples or whether the same sample was measured repeatedly
☐	☒	The statistical test(s) used AND whether they are one- or two-sided<br>*Only common tests should be described solely by name; describe more complex techniques in the Methods section.*
☐	☒	A description of all covariates tested
☐	☒	A description of any assumptions or corrections, such as tests of normality and adjustment for multiple comparisons
☐	☒	A full description of the statistical parameters including central tendency (e.g. means) or other basic estimates (e.g. regression coefficient) AND variation (e.g. standard deviation) or associated estimates of uncertainty (e.g. confidence intervals)
☐	☒	For null hypothesis testing, the test statistic (e.g. F, t, r) with confidence intervals, effect sizes, degrees of freedom and P value noted<br>*Give P values as exact values whenever suitable.*
☒	☐	For Bayesian analysis, information on the choice of priors and Markov chain Monte Carlo settings
☐	☒	For hierarchical and complex designs, identification of the appropriate level for tests and full reporting of outcomes
☐	☒	Estimates of effect sizes (e.g. Cohen's d, Pearson's r), indicating how they were calculated

Our web collection on statistics for biologists contains articles on many of the points above.

Software and code

Policy information about availability of computer code

Data collection	We used EPrime 2.0 (Psychology Software Tools) to present the stimuli in this study.
Data analysis	All data analysis were performed using custom MATLAB R2018b (Mathworks) scripts, which are available here (https://osf.io/cpwx2/). The stochastic tests of transitivity were performed using Q-Test 2.1, a publicly available statistical analysis package for MATLAB (http://regenwetterlab.org/qtest-2-1/).

For manuscripts utilizing custom algorithms or software that are central to the research but not yet described in published literature, software must be made available to editors and reviewers. We strongly encourage code deposition in a community repository (e.g. GitHub). See the Nature Research guidelines for submitting code & software for further information.

Data

Policy information about availability of data

All manuscripts must include a data availability statement. This statement should provide the following information, where applicable:

- Accession codes, unique identifiers, or web links for publicly available datasets
- A list of figures that have associated raw data
- A description of any restrictions on data availability

The dataset analyzed during the current study is available at the Center for Open Science repository at the following link: https://osf.io/cpwx2/

Field-specific reporting

Please select the one below that is the best fit for your research. If you are not sure, read the appropriate sections before making your selection.

- [ ] Life sciences
- [x] Behavioural & social sciences
- [ ] Ecological, evolutionary & environmental sciences

For a reference copy of the document with all sections, see nature.com/documents/nr-reporting-summary-flat.pdf

Behavioural & social sciences study design

All studies must disclose on these points even when the disclosure is negative.

Study description	The study involved quantitative measurement of decision-making behavior. Participants made pairwise decisions between items within different categories (art, chocolate bar brands, gambles) over 2 sessions.
Research sample	The study included individuals with focal brain lesions, in two different groups: those with lesions centered on the ventromedial frontal lobes (VMF; mean age = 59), and those with frontal lobe lesions outside of the VMF (frontal controls; mean age = 66). We also included healthy controls that were matched in age and education (mean age = 62). The lesion patients are recruited based on availability, and the control group is chosen to match the lesion groups, and therefore may not be representative of the general population. The rationale for the sample chosen is that we were interested in the causal role of the VMF in decision-making in humans, and therefore we needed to recruit people with specific damage to that area. The inclusion of the frontal control group was to control for any behavioral effects of brain lesions in the frontal lobe outside of the VMF, and the inclusion of the healthy control group was to control for behavioral effects arising from reasons of age or education.
Sampling strategy	We recruited from the focal lesion databases at the University of Pennsylvania and at the Montreal Neurological Institute (McGill University). As lesions to the VMF are very rare, and very few institutions possess a focal lesion database for research, we recruited from those two institutions in order to obtain the sample sizes, which reflect the maximal number of individuals with brain lesions we are able to recruit within the study period. The sample sizes are in line with most other human lesion studies that involve a group design in the field (eg. Pelletier & Fellows 2019). We also recruited the healthy controls from the healthy control databases from both institutions so that locale differences can be accounted for.
Data collection	We used a laptop computer with EPrime software to present the stimuli to participants. Due to the nature of data collection from individuals with lesions, the study took place either at a site in the institution (University of Pennsylvania or Montreal Neurological Institute) or at the individual's home. However, nobody besides the researcher was present in the immediate vicinity of the experiment (even if there were family members present within the home). Neither the participants nor their family members were aware of the study hypothesis during the study.
Timing	The study took place from December 2014 to January 2016.
Data exclusions	One subject was excluded from the analyses due to the placement of their lesion being too posterior to be considered frontal lobe. For the drift diffusion modeling analysis, 3 subjects (2 healthy controls, 1 VMF) were excluded due to having too many unbreakable ties in their preference ranking of the stimuli. Those participants were included in all of the other analyses.
Non-participation	One subject finished one session of the study, but could not be scheduled for a second.
Randomization	The participants were grouped according to their lesion status (VMF, frontal control, or healthy controls). The intention of the two control groups were to control for factors relating to age, education, and effect of non-VMF frontal lobe lesions.

Reporting for specific materials, systems and methods

We require information from authors about some types of materials, experimental systems and methods used in many studies. Here, indicate whether each material, system or method listed is relevant to your study. If you are not sure if a list item applies to your research, read the appropriate section before selecting a response.

Materials & experimental systems

n/a	Involved in the study	
x		Antibodies
x		Eukaryotic cell lines
x		Palaeontology and archaeology
x		Animals and other organisms
	x	Human research participants
x		Clinical data
x		Dual use research of concern

Methods

n/a	Involved in the study	
x		ChIP-seq
x		Flow cytometry
x		MRI-based neuroimaging

Human research participants

Policy information about studies involving human research participants

Population characteristics	VMF group: 13 participants (7 female), mean age 59.<br>Frontal control group: 10 participants (7 female), mean age 66.<br>Healthy control group: 20 participants (15 female), mean age 62.
Recruitment	Subjects were recruited through the focal lesion databases and healthy control databases at the University of Pennsylvania and Montreal Neurological Institute. Participants self-selected to be a part of the databases, which is true for both healthy controls and for individuals with brain lesions, but we are unaware of any biases that could result for our behavioral results that would not be accounted for with the control groups.
Ethics oversight	The study was approved by both the institutional review boards of the University of Pennsylvania and McGill University.

Note that full information on the approval of the study protocol must also be provided in the manuscript.
